# Supplementary figures and images for: Immunocapture of cell surface proteins embedded in HIV envelopes uncovers considerable virion genetic diversity associated with different source cell types
Source: PLoS One. 2024 Feb 27;19(2):e0296891. doi: 10.1371/journal.pone.0296891 (PMC10898758; doi:10.1371/journal.pone.0296891)

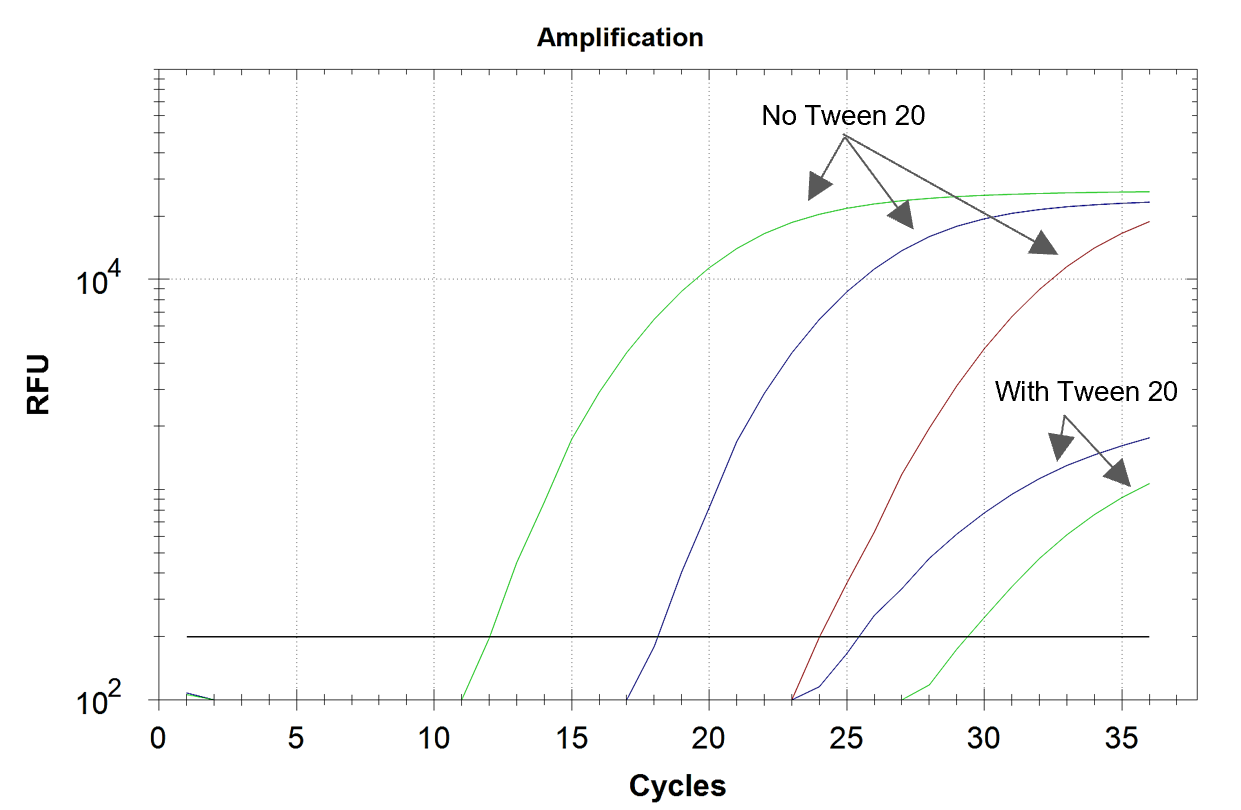

Supplement: S1 Fig — RNA amplifications shown for anti-HLA-DR columns (green lines) and anti-CD16 columns (blue lines), and column with no antibody present (red line). Curves shown for columns either washed three times without Tween 20 or washed with buffer containing 1% Tween 20 as per protocol. Amplification from the no-Ab column (red line) demonstrates particles are non-specifically retained on columns in the absence of Tween 20. (TIF) [file pone.0296891.s001.tif]

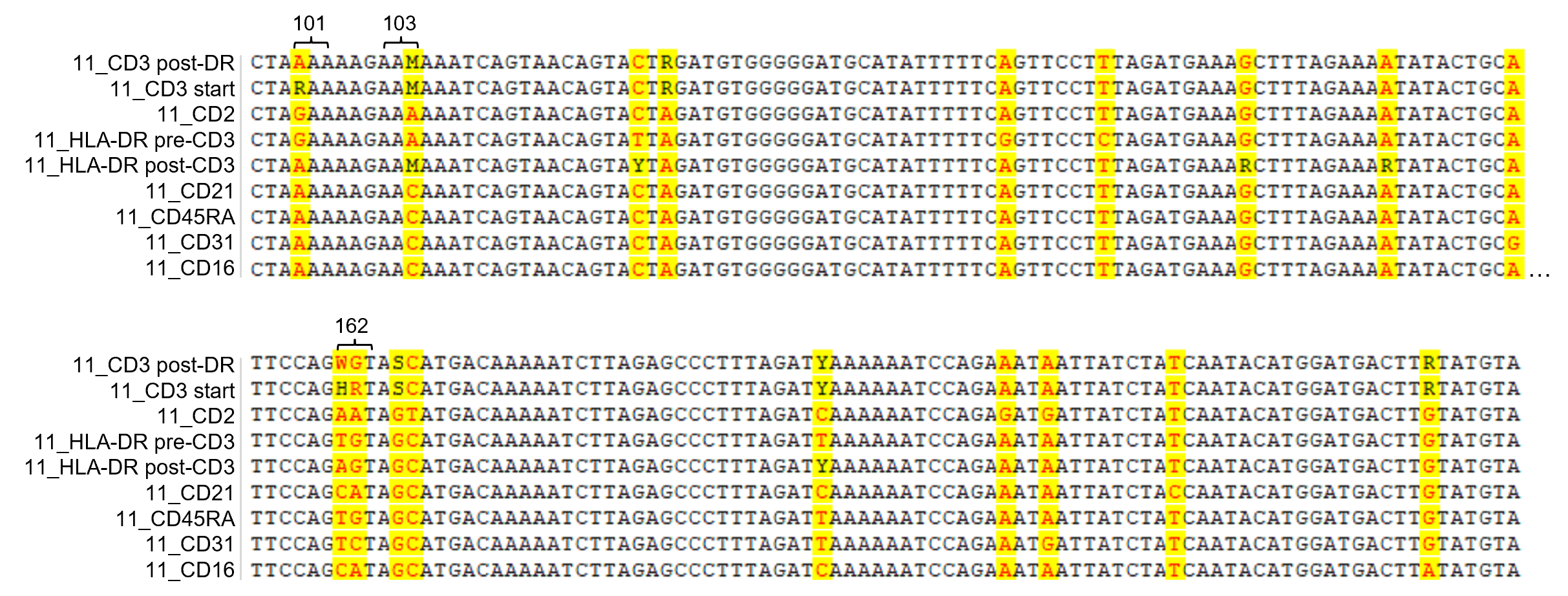

Supplement: S2 Fig — The upper and lower alignments represent two regions in HIV-1 reverse transcriptase (RT) from an individual who was administered intrapartum SD-NVP. Two series of captures are demonstrated in the alignments. In the initial capture order the anti-CD3 capture was placed after anti-HLA-DR (CD3 post-DR), then on reanalysis the CD3 capture was placed at the beginning of the algorithm (CD3 start). The “CD3 start” would have comprised a broad representation of T cell-derived virion, whereas placed after HLA-DR the CD3 capture would have represented non-activated, mature T cell sources. RT amino acid codon positions are indicated above. Codon 101 (A→G) and 103 (A→C) mutations confer NVP resistance and were differentially selected among virions associated with different CD markers as were background polymorphisms (e.g., codon 162). (TIF) [file pone.0296891.s002.tif]

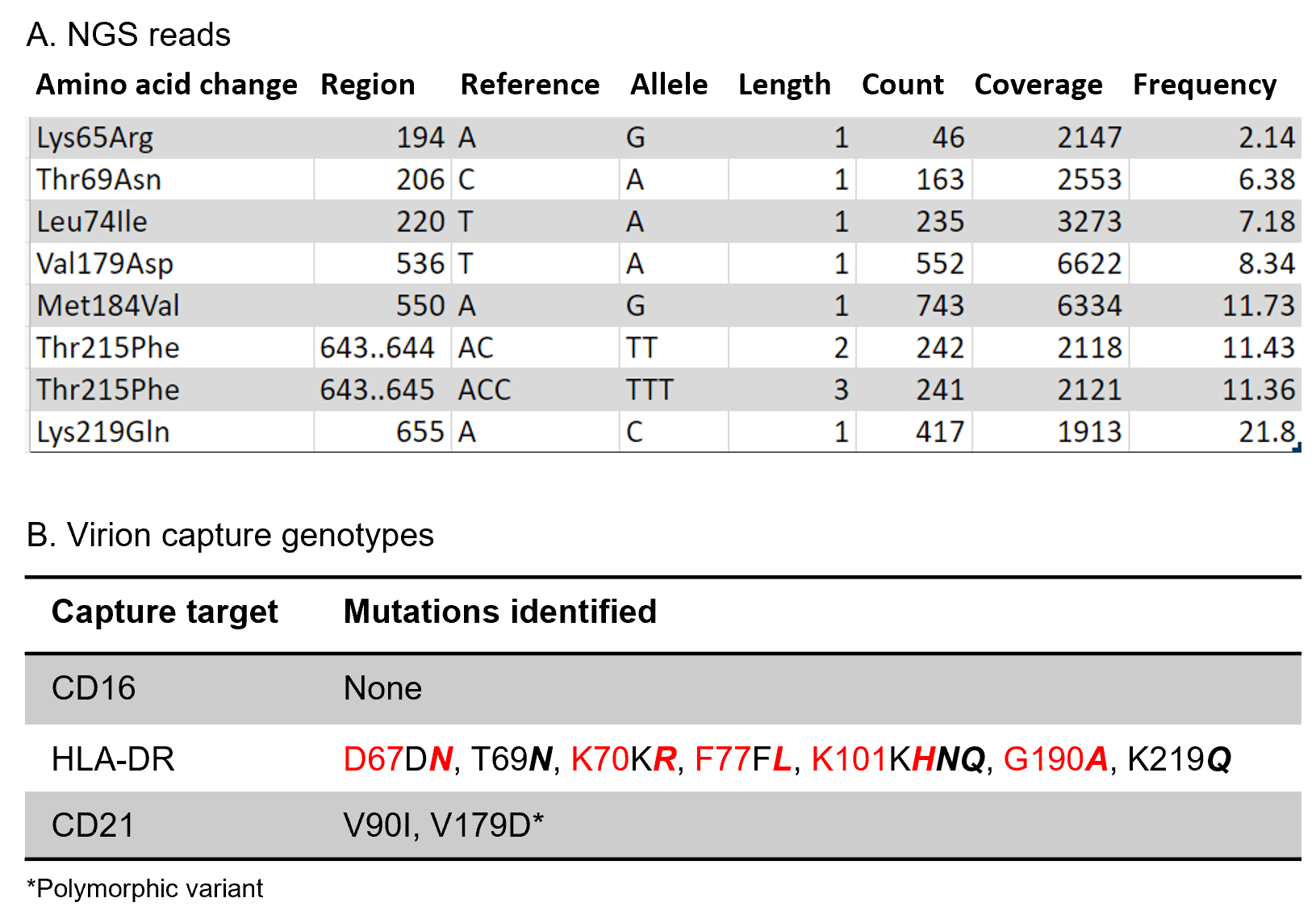

Supplement: S3 Fig — Comparison of HIV mutations at codons associated with drug resistance identified by next-generation sequencing (NGS) (A.) and those obtained from virion particle captures (B.) from the early-acute sample ID 9012 collected at 12 days pre-seroconversion. A. Trimmed NGS reads were mapped to HXB2 reverse transcriptase (RT) reference sequence followed by the removal of any duplicate mapped reads. Variants above a 2% frequency of total read coverage are included. B. Captured drug resistance mutations at this timepoint that were not identified by NGS (in red) were all later identified by captures at 5 days pre-seroconversion associated with myeloid populations. (TIF) [file pone.0296891.s003.tif]
