# Supplementary material for: Immunocapture of cell surface proteins embedded in HIV envelopes uncovers considerable virion genetic diversity associated with different source cell types
Source: PLoS One. 2024 Feb 27;19(2):e0296891. doi: 10.1371/journal.pone.0296891 (PMC10898758; doi:10.1371/journal.pone.0296891)
Supplement: S1 Table — (DOCX) [file pone.0296891.s004.docx]

**Table S1.** Test of percent Tween 20 on non-specific retention of virus on columns without antibody.

| Percentage  Tween 20 | Column extract | Flow through |
| --- | --- | --- |
| 0.1% | Detected | Detected |
| 0.2% | Detected | Detected |
| 0.4% | Not detected | Detected |
| 1.0%^‡^ | Not detected | Detected |

Buffer formula evaluated: PBS + 1% fetal bovine serum or bovine serum albumin (BSA) + [x%] Tween 20. BSA was found to yield cleaner columns with the no-Ab tests and was used for this table evaluation. ^‡^Concentration used for washes and block that retained mAb capture capability. Higher concentration selected to alleviate viscous samples.
