# Supplementary material for: Immunocapture of cell surface proteins embedded in HIV envelopes uncovers considerable virion genetic diversity associated with different source cell types
Source: PLoS One. 2024 Feb 27;19(2):e0296891. doi: 10.1371/journal.pone.0296891 (PMC10898758; doi:10.1371/journal.pone.0296891)
Supplement: S2 Table — (DOCX) [file pone.0296891.s005.docx]

**Table S2.**  Monoclonal antibodies used in capture evaluations.

| **Antibody**  **target** | **Clone** |
| --- | --- |
| CD2 | MT910 |
| CD3 | UCH-T1 |
| CD10 | F-4 |
| CD11c | B-6 |
| CD14 | 61D3 |
| CD15 | By87a |
| CD16 | 2Q1240 |
| CD21 | A-3 |
| CD27 | H-260 |
| CD31 | M-185 |
| CD36 | SMφ |
| CD44 | F-4 |
| CD45-RA | 4KB5 |
| CD45-RO | UCH-L1 |
| CD55 | H-7 |
| CD68 | KP1 |
| HLA-DR | HL-38 |
