# Supplementary material for: Immunocapture of cell surface proteins embedded in HIV envelopes uncovers considerable virion genetic diversity associated with different source cell types
Source: PLoS One. 2024 Feb 27;19(2):e0296891. doi: 10.1371/journal.pone.0296891 (PMC10898758; doi:10.1371/journal.pone.0296891)
Supplement: S1 File — (PDF) [file pone.0296891.s006.pdf]

Immunocapture of virion from body fluids

Jeffrey A. Johnson[1], Sarah Sabour[1], Jin-fen Li[1], Jonathan Lipscomb[1]

[1]CDC

jjohnson

Protocol Info: Jeffrey A. Johnson, Sarah Sabour, Jin-fen Li, Jonathan Lipscomb . Immunocapture of virion from body fluids. **protocols.io** https://protocols.io/view/immunocapture-of-virion-from-body-fluids-c4biyske

Created: Oct 19, 2023

Last Modified: Oct 31, 2023

PROTOCOL integer ID: 90186

DISCLAIMER

The performance of this protocol is claimed by the authors and does not necessarily represent the official view of the CDC.

ABSTRACT

Procedure for immunocapturing HIV virions from blood and seminal plasma, cerebral spinal fluid, and cell culture supernatant by monoclonal antibody-targeting source cell markers in virion envelopes.

ATTACHMENTS

nwqnb9rdx.pdf

GUIDELINES

Workflow Chart:

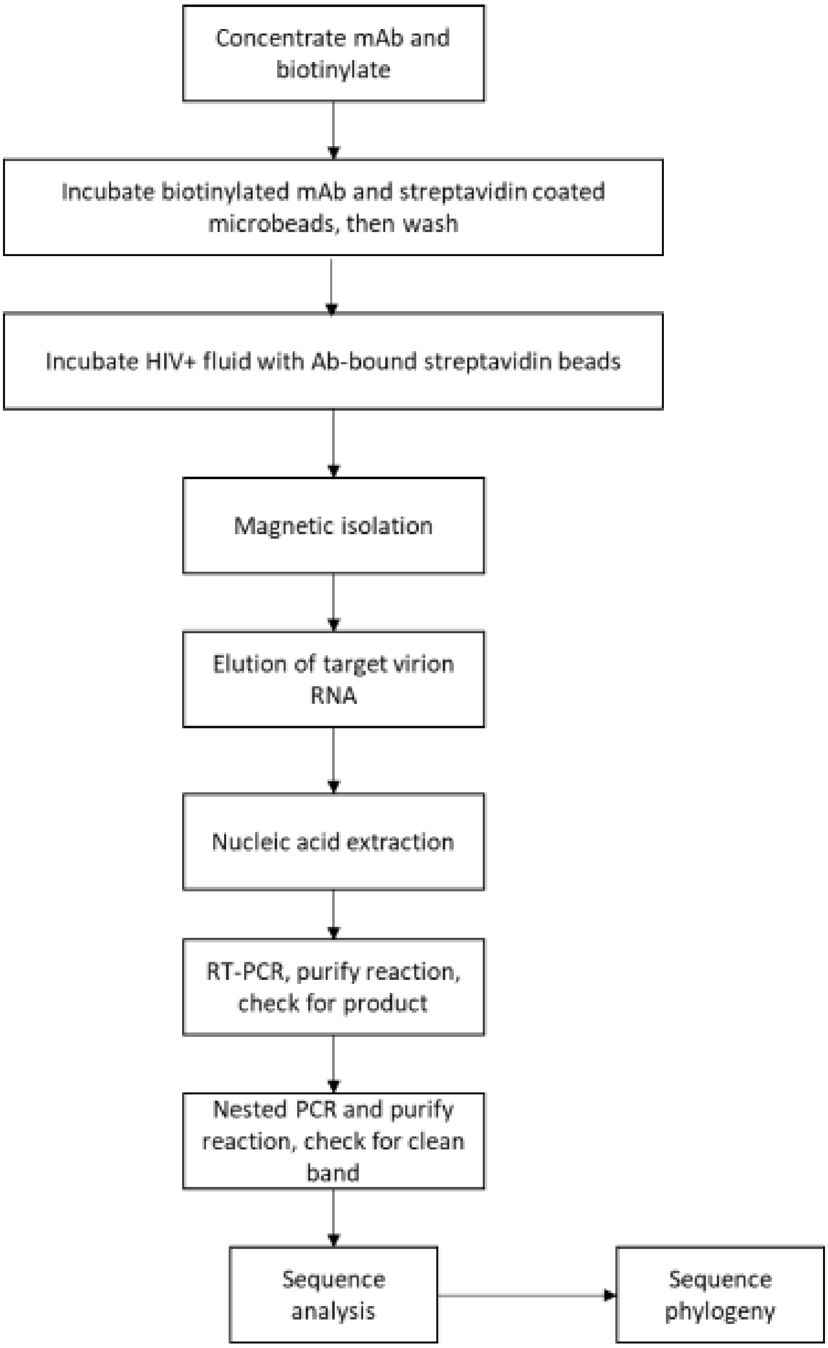

Definitions:

Term	Definition
RT	Reverse Transcription
PCR	Polymerase Chain Reaction
nPCR	Nested PCR

MATERIALS

Equipment:

- Roller-mixer: Stuart SRT9
- Microcentrifuge (e.g., Eppendorf 5415D)
- Template Tamer/CleanSpot workstation
- UV cross-linker
- Thermocycler and real-time cycler (e.g., Bio-Rad OPUS)
- Genetic sequence analyzer

Reagents and Media:

- **Monoclonal antibodies:** sourced from Santa Cruz Biotechnology (SCBT.com)

A	B	C	D	E	F	G
S.No	PRODUCT NAME	CAT. #	ISOTYPE	EPITOPE	APPLICATIONS	SPECIES
1	CD16 (2Q1240)	SC-70548	mouse IgG1	FL (h)	WB,IP,IF,IHC(P),FCM	human
2	CD14 (61D3)	sc-52475	mouse IgG1	Extracellular (h)	IP,IF,IFCM	human
3	PECAM-1/CD31 (158-2B3)	sc-65260	mouse IgG1	FL (h)	WB,IP,IF,FCM	human
4	CD45RA (4KB5)	sc-20057	mouse IgG1	FL (h)	WB,IP,IF,IHC(P),FCM	human
5	CD45RO (UCHL1)	sc-1183	mouse IgG2a	FL (h)	WB,IP,IF,IHC(P),FCM	human
6	HLA-DR/DP (HL-38)	sc-51616	mouse IgG2a	FL (h)	WB,IP,FCM	human
7	CD27 (H-260)	sc-20923	rabbit IgG	FL (h)	WB,IP,IF,ELISA	human>mouse, rat
8	CD3-ε (UCH T1)	sc-1179	mouse IgG1	FL (h)	WB,IP,IF,IHC(P),FCM	human
9	CD2 (MT910)	sc-19638	mouse IgG1	FL (h)	WB,IP,IF,IHC(P),ELISA	human
10	CD21 (A3)	sc-13135	mouse IgG2b	AA 21-260 (h)	WB,IP,IF,IHC(P),ELISA	mouse, human
11	Integrin αX/CD11c (B6)	sc-46676	mouse IgG1	FL (h)	WB,IP,IF,IHC(P),ELISA	human
12	Iba1 (F-4)	sc-398406	mouse IgG1	FL (h)	WB,IP,IF,IHC(P),ELISA	human
13	CD36 Antibody (SMφ)	sc-7309	mouse IgM κ	Extracellular (h)	WB, IP, IF, IHC(P), FCM	mouse, rat and human
14	CD68 (KP1)	sc-20060	mouse IgG1	Extracellular (h)	WB, IP, IF, IHC(P) and FCM	mouse, rat and human

CD16 Antibody (2Q1240) **Santa Cruz Biotechnology Catalog #sc-70548**

CD14 Antibody (61D3) **Santa Cruz Biotechnology Catalog #sc-52457**

CD31/PECAM-1 Antibody (158-2B3) **Santa Cruz Biotechnology Catalog #sc-65260**

- CD45RA Antibody (4KB5) **Santa Cruz Biotechnology Catalog #sc-20057**
- CD45RO Antibody (UCH-L1) **Santa Cruz Biotechnology Catalog #sc-1183**
- HLA-DR/DP Antibody (HL-38) **Santa Cruz Biotechnology Catalog #sc-51616**
- CD3-ε Antibody (UCH-T1) **Santa Cruz Biotechnology Catalog #sc-1179**
- CD2 Antibody (MT910) **Santa Cruz Biotechnology Catalog #sc-19638**
- CD21 Antibody (A-3) **Santa Cruz Biotechnology Catalog #sc-13135**
- Integrin αX/ITGAX/CD11c Antibody (B-6) **Santa Cruz Biotechnology Catalog #sc-46676**
- Iba1 Antibody (F-4) **Santa Cruz Biotechnology Catalog #sc-398406**
- CD36 Antibody (SMφ) **Santa Cruz Biotechnology Catalog #sc-7309**
- CD68 Antibody (KP1) **Santa Cruz Biotechnology Catalog #sc-20060**
- BiotinTag Micro Biotinylation kit (BTAG), Sigma BTAG-1KT
- Dimethyl sulfoxide **Merck MilliporeSigma (Sigma-Aldrich) Catalog #D5879**
- Bicinconinic acid kit, Sigma BCA1-1KT
- µMACS Streptavidin MicroBeads, Miltenyi 120-001-017
- Equilibration Buffer for nucleic acid applications, Miltenyi 120-001-014
- 20 µMACS Columns: Miltenyi 120-001-002
- PBS 0.01M pH7.4, CDC #4550
- Tween 20 100% Nonionic Detergent **Bio-Rad Laboratories Catalog #1706531**
- Bovine Serum Albumin **Merck MilliporeSigma (Sigma-Aldrich) Catalog #A9418**
- Wash buffer: PBS +1% BSA + 1% Tween 20
- Blocking buffer: PBS +1% BSA + 1% Tween 20
- Ethanol (96 – 100%)
- DEPC-Treated Water **Thermo Fisher Catalog #AM9906**
- 0.1 M Sodium Phosphate Buffer, pH 7.2, Sigma P9693
- QIAamp® Viral RNA Mini **Qiagen Catalog #52906**

- QIAquick PCR Purification Kit **Qiagen Catalog #28104**
- Qubit™ dsDNA HS Assay Kit **Invitrogen - Thermo Fisher Catalog #Q32851** / Qubit® dsDNA HS Assay Kit **Thermo Fisher Scientific Catalog #Q32854**
- Elution buffer: 0.01 M Tris-Cl in DEPC-treated water
- BigDye XTerminator™ Purification Kit **Thermo Fisher Catalog #4376486**
- SuperScript**TM** III RT/ Platinum**TM** Taq HiFi: Invitrogen 12574
- RNase Inhibitor **Thermo Fisher Catalog #N8080119**
- Platinum™ SuperFi II PCR Master Mix **Invitrogen - Thermo Fisher Catalog #12368050**
- DNase DNA-free kit, Invitrogen AM1906 (for tissue culture supernatants)

Supplies, Other Materials:

Equipment	
**Amicon Ultra-0.5 Centrifugal Filter Unit**	NAME
Centrifugal Filter Unit	TYPE
Millipore	BRAND
UFC5003BK	SKU
https://www.merckmillipore.com/GB/en/product/Amicon-Ultra-0.5-Centrifugal-Filter-Unit,MM_NF-UFC5003BK	LINK

- Magnetic Separator: 8 position MACS magnetic stand 007139
- Sterile, RNase-free microcentrifuge tubes, 1.5 mL – 2 mL
- 10 µL, 200µL, 1000µL pipette and tips
- RNase-free pipet tips with aerosol barrier
- Immulon II flat well 96-well plates, Nunc #96920
- Microcentrifuge tube racks
- Clear microfilm seals for plates
- 96-well hard-shell skirted conical bottom PCR plates
- 96-well non-skirted clear conical bottom sequencing plates
- 96-well septa mats
- Dedicated spaces for reagent preparation, RNA template, PCR/nested PCR, Real-Time PCR, and sequencing. Gloves must be changed as needed to prevent template contamination.

Sample Information / Processing (Volume, labeling, handling, storage)

- Fresh, non-frozen biologic sample preferred, stored at 4 °C and used within 48 hours. If frozen, thaw frozen plasma On ice .
- Aliquot desired input plasma volume from 200 µL – 400 µL , equivalent to <= 500,000 virus copies, into a 2 mL microcentrifuge tube.

Note

If viral load is unknown, determine copies by qPCR or test on a commercial viral load platform.

Concentrate Antibody

32m

1 Use Amicon Ultra-0.5 Centrifugal Filter Devices.

2 Insert the Amicon device into the microcentrifuge tube.

3 Add up to 500 µL Ab (0.1 undetermined - 0.2 undetermined) to the filter device and cap it.

4 Insert the capped Amicon Ultra device into a centrifuge tube and place in the centrifuge rotor.

5 Spin the device at 14000 x g, 00:30:00 .

30m

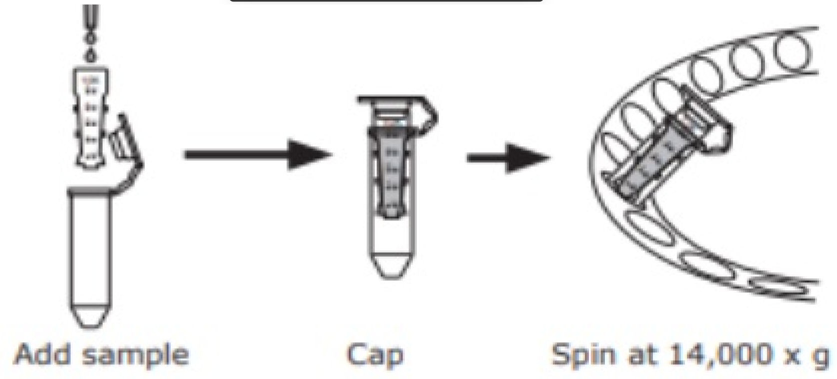

6 Remove the device and place it upside down in a clean tube, place in centrifuge, aligning the open cap strap, toward the center of the rotor.

7 Spin the device at 1000 x g, 00:02:00 .

2m

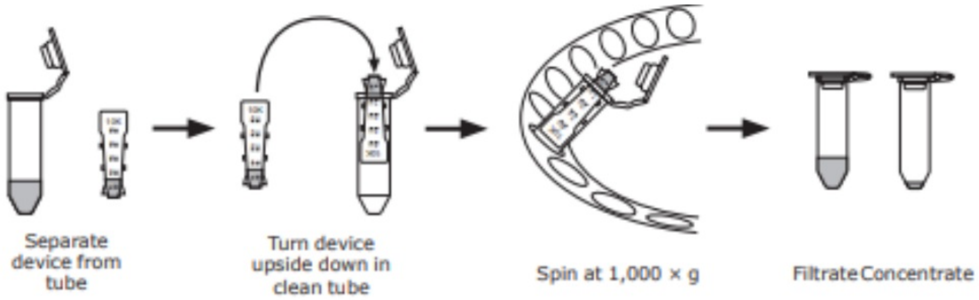

8 Add SPB ([M] 0.1 Molarity (M) sodium phosphate buffer, pH 7.2) to achieve a final volume 100 µL mAb at a concentration of 1 undetermined - 2 undetermined .

Antibody Biotinylation (Sigma BTAG)

2h 30m

9 Add 30 µL DMSO to the vial of Biotinylation Reagent (BAC-SulfoNHS), and then add 970 µL [M] 0.1 Molarity (M) sodium phosphate buffer.

Note

The concentration of Biotinylation Reagent is 5 undetermined .

10 Immediately add 2 µL of Biotinylation Reagent to the antibody solution with gentle stirring.

11 Incubate with gentle stirring for 00:30:00 at Room temperature or 02:00:00 at 2 °C - 8 °C . 2h 30m

Isolation of Labeled Antibody (Sigma BTAG)

6m

12 Place the column G-50 in a 1.5 ml Eppendorf tube, pre-spin the column for 00:01:00 at 700 x g (3000 rpm). 1m

13 Add 200 µL PBS (pH 7.4) to the column, spin the column for 00:01:00 at 700 x g (3000 rpm). 1m

14 Repeat two times.

15 Label two of 1.5 ml Eppendorf tube.

16 Place column in tube 1 and apple the biotinylation reaction mix to the column.

17 Centrifuge the column for 00:02:00 at 700 x g and collect flow-through (fraction 1). 2m

18 Place column in tube 2 and add 200 up to the column, spin the column for 00:02:00 at 700 x g . collect flow-through (fraction 2). 2m

Determine Ab Concentration

30m

19 Use Bicinchoninic Acid Kit, 96 well Immulon II plate assay.

20 Prepare standard curve dilutions:

A	B	C	D
Protein Ci (µg/mL)	Protein Input Volume (uL)	PBS (µL)	Protein Cf (ug/mL)
1000	-	-	1000
1000	400	100	800
800	375	125	600
600	333	166	400
400	250	250	200
200	250	250	100

21 Prepare BCA Working Reagent: Mix Reagent A(50) and Reagent B(1).

22 Add 25 µL protein standard solution, PBS, and Ab samples into well of 96 well plate. Duplicate.

23 Add 200 µL of BCA working to each well (1:8 protein/BCA ratio).

24 Cover the plate with film and incubate 37 °C for 00:30:00. 30m

25 Read the absorbance at 562 undetermined (540 undetermined - 590 undetermined).

26 Calculate mAb concentration against the standard curve.

ELISA to Check Biotinylated Antibody

2h 45m

27 Using Immulon II 96 well plate.

28 Coat three wells with a dilution series of mAb beginning with 1 µL mAb in 99 µL PBS (1:100) continuing with two more 10-fold dilutions. Incubate Overnight at 4 °C. 30m

29 Wash plate 4 times with PBS+0.05% Tween.

30 Add 100 µL blocking buffer to each well, incubate at 37 °C for 01:00:00. 1h

31 Wash plate 4 times with PBS+0.05% Tween.

32 Add 100 µL of 1:5000 ExtrAvidin_Peroxidase diluted with blocking buffer to each well. Cover 1h

plate and incubate at 37 °C for 01:00:00 .

33 Wash plate 4 times with PBS+0.05% Tween.

34 Add 100 µL TMB substrate to each well.

35 Develop plate at Room temperature in the dark for 00:15:00 . 15m

36 Add 100 µL of stop solution to each well.

37 Read the absorbance of each well at 450 undetermined and 550 undetermined . OD values of the 1:100 dilution (first well) of ≥0.6 indicates adequate biotin labelling of antibody.

Streptavidin coated beads_ Biotinylated Ab + HIV → bead-Ab_... 1h 18m

38 Dilute Biotinylated Ab to 0.4 undetermined with [M] 0.1 Molarity (M) sodium phosphate buffer.

39 Incubate 100 µL of Streptavidin coated beads with 5 µL PBS (negative Ab control) or 2 µg (5 µL of 0.4 undetermined) biotinylated Ab for 00:10:00 at Room temperature on a roller platform. 10m

40 Centrifuge bead-Ab complex at 8000 rpm, 00:10:00 . 10m

41 Remove supernatant and wash pellet with 100 µL PBS+1% BSA +1% Tween 20, centrifuge beads Ab complex at 8000 rpm, 00:10:00 . Wash 3 times. 10m

42 Add 100 µL Blocking buffer (PBS+1% BSA +1% Tween 20) to the tube and incubate at 10m

4 °C Overnight .

43 Centrifuge bead-Ab complex at 8000 rpm, 00:08:00 and then remove supernatant. 8m

44 If working with tissue culture supernatants first DNase treat and inactivate.

45 Add 200 µL HIV-positive material (plasma, CSF, Semen, Culture or flow-through) to the designated bead-Ab complex and incubate for 00:30:00 at Room temperature . Mixing gently on a roller-mixer. 30m

Prepare µMACs column

46 Attach µMACs column to the magnetic multistand.

47 Add 100 µL equilibration buffer for nucleic acid applications to the column.

48 Rinse column with 100 µL wash buffer (PBS+1% BSA +1% Tween 20), twice.

Binding HIV-bead-Ab complex to the column and collecting th... 30m

49 Apply HIV-bead-Ab complex onto the top of column, collecting the flowthrough in a clean microfuge tube or eluting directly into the next tube of biotinylated mAb-bead complex. Let reaction pass through the column completely, captured virus will be retained on the column and flow-through will contain non-target virus (see figure below).

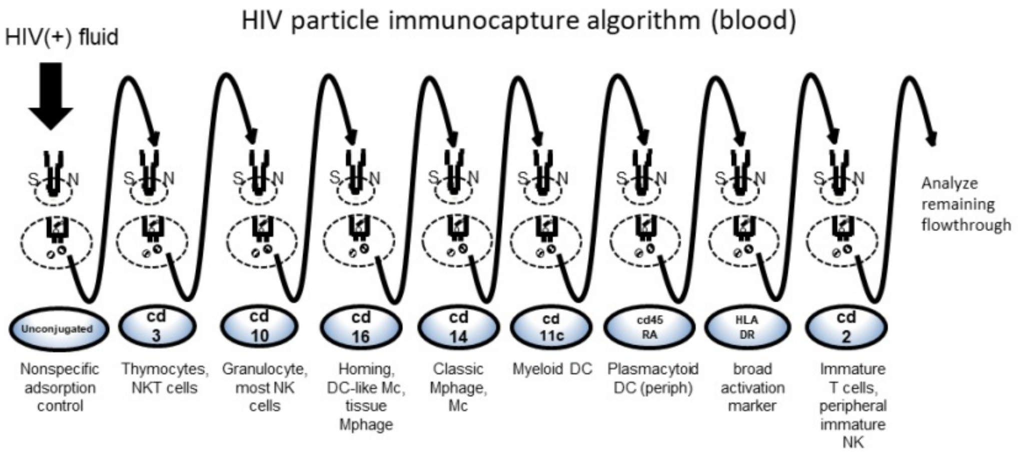

50 Add 30 µL wash buffer to the column and collect the flow.

Note

This accounts for the column void volume and maintains a 200 µL sample volume.

51 Incubate the flowthrough with next mAb-bead complex for 00:30:00 on the roller-mixer at Room temperature . 30m

52 To the just-eluted column, rinse the column 3 times with 400 µL of wash buffer to remove nonspecifically bound material, allowing the column drain completely. Discard the wash.

53 Repeat this process until all mAb-bead columns in the series are completed.

Elute target virion RNA from the column (using the QIAamp V... 10m

54 After washing the column, place the column of bound virion in a new 1.5 mL Eppendorf tube.

55 Add 50 µL AVL lysis buffer to the column and pass through the column completely.

56 Add another 150 µL AVL lysis buffer to the column and pass through the column completely.

57 Add 360 µL AVL lysis buffer to the tube of eluted lysate and incubate tube at Room temperature for 00:10:00. Continue with the extraction kit instructions as follows. 10m

Note

Take 140 µL of final flow through after all columns are completed and add 560 µL of lysis buffer. Continue with lysis kit steps.

RNA extraction: QIAamp Viral RNA Mini Kit

8m 15s

58 Add 560 µL ethanol (96–100%) to the sample and mix by pulse-vortexing for 00:00:15. After mixing, briefly centrifuge the tube to remove drops from inside the lid. 15s

59 Carefully apply 630 µL of the sample solution to the QIAamp Mini column (in a 2 ml collection tube) without wetting the rim. Close the cap, and centrifuge at 6000 x g (8000 rpm) for 00:01:00. Place the QIAamp Mini column into a clean 2 ml collection tube and discard the tube containing the filtrate. 1m

60 Repeat this step until all of the lysate has been loaded onto the spin column.

61 Add 500 µL Buffer AW1. Close the cap, and centrifuge at 6000 x g (8000 rpm) for 00:01:00. Place the QIAamp Mini column in a clean 2 ml collection tube. 1m

62 Add 500 µL Buffer AW2. Close the cap and centrifuge at full speed (20000 x g; 14000 rpm) for 00:03:00. 3m

63 Place the QIAmp Mini column in a new 2 mL collection tube and discard the old collection tube 1m

with the filtrate. Centrifuge at 20000 x g (full speed) for 00:01:00.

64 Place the QIAamp Mini column in a clean 1.5 ml microcentrifuge tube. Discard the old collection tube containing the filtrate. Carefully open the QIAamp Mini column and add 60 µL Buffer AVE equilibrated to Room temperature.

65 Close the cap and incubate at Room temperature for 00:01:00. Then centrifuge at 6000 x g (8000 rpm) for 00:01:00. 2m

RT PCR: SuperScript™ III One-Step RT-PCR System with Platin...

66 Thaw, vortex briefly to mix and centrifuge each component before use.

67 Prepare 45 µL reaction mast mix in a PCR workstation.

A	B
Component	Volume (uL)
2x Reaction Mix	25
F primer (10 µM)	1
R primer (10 µM)	1
SuperScript III RT/Platinum Taq High Fidelity Enzyme Mix	2
RNA Inhibitor (40 U/µL)	1
Water	15
Total	45

68 Add 5 µL of template RNA. Final reaction volume is 50 µL.

69 Gently mix and make sure that all the components are at the bottom of the amplification tube.

70 Place the reaction in the preheated thermal cycler programmed as described above. Collect the data and analyze the results.

71 Program the thermal cycler to amplify with the following conditions:

1x			40x			1x
25 °C	55 °C	94 °C	94 °C	55 °C	68 °C	68 °C
10 minutes	30 minutes	2 minutes	2 minutes	30 seconds	1 minute	5 minutes

Note

You may check for primary PCR product by gel electrophoresis or real-time detection. Due to the potential for low copy numbers perform nested reactions.

Nested PCR (nPCR): Platinum™ SuperFi II PCR Master Mix

72 Thaw, vortex briefly to mix and centrifuge each component before use.

73 For each sample, prepare 48 µL reaction master mix in a PCR workstation as follows:

A	B
Component	Volume (uL)
Platinum SuperFi II PCR Master Mix	25
F primer (10 µM)	1
R primer (10 µM)	1
Water	21
Total	48

74 Transfer new reaction microfuge tubes and RT-PCR samples to Nested PCR room. Add 2 µL of each RT-PCR sample per tube.

75 Use a designated 2nd round PCR thermocycler – vortex and quick spin samples before inserting into thermocycler. Amplify with the following conditions (specific for primers used):

1x<br>98 °C<br>2 minutes	30x<br>98 °C<br>15 seconds	 56 °C<br>30 seconds	 68 °C<br>1 minute	1x<br>68 °C<br>5 minutes

76 DNA is quantified and PCR amplicon size is verified via the Agilent 2200 Tapestation after nested PCR is performed for sequencing. Alternatively, bands can be checked by agarose gel.

77 Identify samples with clean amplicon bands for further analysis.

78 **Perform Sanger sequencing with available platform.**

Sequence analysis

79 Compare relatedness of HIV sequences in alignment software (e.g., Geneious) and MEGA to generate neighbor-joining trees and perform genetic distance analysis. Perform best model fit (typically Tamura 92 is the best fit)

Sample Retention and Storage

80 Note

- Frozen plasma specimens should be stored at -80 °C until ready for testing.
- Extracted genetic material should be stored at -80 °C for long-term storage.
- Amplified RT-PCR can be stored for two weeks at 4 °C but should be stored at -80 °C for longer storage.
- RT-PCR amplicons should not be stored with clinical samples.
