# Supplementary material for: Immunocapture of cell surface proteins embedded in HIV envelopes uncovers considerable virion genetic diversity associated with different source cell types
Source: PLoS One. 2024 Feb 27;19(2):e0296891. doi: 10.1371/journal.pone.0296891 (PMC10898758; doi:10.1371/journal.pone.0296891)
Supplement: Attachment — Submitted filename: PlosOne 3 recommendations_Togre N.pdf [file pone.0296891.s007.pdf]

Reviewer comment for Editor and authors

I appreciate the opportunity to review the manuscript entitled " Immunocapture of Cell Surface Proteins Embedded in HIV Envelopes Uncovers Considerable Virion Genetic Diversity Associated with Different Source Cell Types". In the current study, Sarah Sabour and colleagues have evaluated the virion genetic diversity possibly associated with the different source types through the HIV immunocapture method. The same authors have designed various deductive algorithms using mAbs based on cellular markers. Authors have reported distinct variants expressed within individuals with drug resistance mutations undetected by sequencing and often associated with markers indicative of myeloid lineage.

However, the author may take note of the major and minor remarks listed below to improve the manuscript:

I have major concerns about discussion and methodology. I would encourage authors to rewrite the discussion with more references, as in the current state it seems to be just observations. Also, though the article type is laboratory protocol, I recommend adding more references in the methodology section as these protocols are complex.

**Abstract:**

The abstract is written very well, and it provides a concise overview of the study's objectives and findings.

**Introduction:**

The introduction needs to be refined, as it is very short on study background. It should effectively introduce the key concepts, research gaps, and objectives of the study.

Here are a few suggestions for improvement:

- I kindly request that authors emphasize how HIV persistence and drug resistance arise based on drug permeability and cell lineages.
- What are the major ‘research gaps’ that lead to the study hypothesis?

**Material and methods:**

This section provides a solid foundation for conducting the study. However, in this article, some areas could be further improved to enhance clarity and reproducibility.

- As mentioned earlier, at certain points, there is a need to add more references. For example, Line No. 82,
- I am not able to find the number of samples used in this study. Please mention them in this section.
- Is there any way to include more information about wash buffer formulation as the whole protocol and study are based on this phenomenon?
- Please remove the section that starts from line 153 and keep it at the start of the methodology section.
- The part written from lines 161 to 171 can be shortened as it seems redundant to some parts of the introduction.
- Does the use of lysis buffer on the column to recover RNA hamper the column's capacity to reuse it again for the next elution?

**Results:**

The overall result section is written very well.

- Please change figure 1.
- Also, the authors have given the cell sorting data as supplementary. However, I personally feel putting these results in a manuscript will strengthen the study hypothesis and the value of the manuscript.

Discussion:

- As mentioned earlier, I recommend adding more references to strengthen the observed results. The conclusion needs to be rewritten as it lacks conciseness about the observed parameters and the importance of the study.
- There is scope for shortening the summary.
